# Reply to Letter to the Editor: “Trans-arterial embolization for treatment of acute lower gastrointestinal bleeding—a multicenter analysis”

**DOI:** 10.1007/s00330-025-11488-2

**Published:** 2025-03-06

**Authors:** Timo A. Auer, Bernhard Gebauer

**Affiliations:** 1 Department of Radiology, Charité – Universitätsmedizin Berlin, Corporate Member of Freie Universität and Humboldt-Universität zu Berlin, Charitéplatz 1, 10117 Berlin, Germany; 2 Berlin Insitute of Health at Charité-Universitätsmedizin Berlin, Charitéplatz 1, 10117 Berlin, Germany

We sincerely thank Romaric Loffroy (R.L.) for his valuable and insightful comments on our study. His observations not only validate but also challenge our multicentric study and confirm our statement in the “Discussion” section: “… there has been an ongoing debate regarding the most appropriate and safest embolic agent or device. The heated discussion is driven by concerns about potential successive infarctions of intestinal segments, which might necessitate surgical intervention” [1].

First, we would like to address the comments regarding the frequency of complications related to ischemia. We were equally surprised by the higher frequency compared to published literature. We attribute this primarily to the large number of critically unstable patients, where it may not always be possible to fully differentiate between the effects of embolization and other factors, such as hemorrhagic infarction or the consequences of sepsis. In our study, we reported the post-embolization surgical resection rate, and it is possible that the post-embolization ischemia rate was overestimated [1].

Given R.L.’s comments on the interventional radiology expertise of the participating centers, we consider all centers’ experience and training as state-of-the-art. However, transarterial embolization (TAE) for lower gastrointestinal bleeding (LGIB) is increasingly more frequent than 10 years ago because it is recommended in recent guidelines among first-line treatments, which is also reflected in our recruitment [1–3]. R.L. rightly points out that the interventionalists’ experience was not reported, and although all centers’ experience is significant, it does not necessarily reflect the skill level of individual interventionalists, which can vary especially during night shifts and on duty (where it can be assumed that a majority of TAE has been performed). Subsequently our study that collected data across five German university centers mirrors the reality of clinical interventional radiology practice. It is important to acknowledge that we are presenting multicenter data on this topic for the first time, further providing a thorough representation of routine practice. Given that the data challenges some current practice guidelines, the results should be taken into account in the formulation of future guidelines [4].

Second, we recognize that there are varying opinions on embolization materials and techniques. However, we believe there is a shared consensus on one key point: embolization should be carried out as selectively and safely as possible. At a superselective level, we consider coil embolization to be the safest method, regardless of experience level. Recent data, including our own, suggest that even when combined cautiously with particles of at least 300 µm, there seems to be no increased risk of ischemia when embolizing selectively [1, 5]. In a recent, retrospective literature review by Ini’ et al including 1019 patients from 32 published studies using TAE in LGIB, the authors highlighted that “Coils are the preferred embolizing material, followed by gelfoam and PVA” and noted that “Glues require changing of catheter after use and experience in release” [6].

Glue, containing cyanoacrylates (CA), is an excellent embolic agent and offers significant advantages, particularly in situations involving compromised coagulation. Furthermore, with the newer cyanoacrylate types like Glubran®2 (N-Butyl CA (NBCA) plus Methacryloxy Sulfolane) and MagicGlue® (N-Hexyl CA (NHCA)) polymerization times are prolonged, reducing the risk for glueing of the catheter tip and non-target embolization. There is growing monocentric and retrospective evidence supporting the use of glue in managing LGIB [7–9]. However, the risk of non-target embolization cannot be eliminated, and even with the newer CA types, there is a learning curve in determining both the depth of glue penetration into the targeted area and the amount required. For this reason, we still recommend that glue should be used by experienced interventional radiologists only, especially in cases of intestinal bleeding. R.L. is currently one of the most experienced users of glue for embolization. However, the use of glue in less experienced hands may be more problematic than the precise placement of perfectly visible (detachable) microcoils. This is reflected by the preferred choice of embolization materials in our study.

In summary, glue (cyanoacrylate) has received more attention in recent years. It is an excellent embolic material, and the newer longer-polymerizing CA types further enhance its use in embolic procedures. Its handling requires expertise and adequate training. It has an advantage in severely impaired coagulation. Nevertheless, for the treatment of GI bleeding, microcoils are preferred in most centers in view of the simplicity, visibility and precision of this technique, as shown by our study.
